# Design and Implementation of a Three-Segment Tendon-Driven Continuum Robot with Variable Stiffness for Manipulation in Confined Spaces

**DOI:** 10.3390/biomimetics11020113

**Published:** 2026-02-04

**Authors:** Zhixuan Weng, Liansen Sha, Yufei Chen, Bingyu Fan, Lan Li, Bin Liu

**Affiliations:** 1 School of Biomedical Engineering (Suzhou), Division of Life Sciences and Medicine, University of Science and Technology of China, Hefei 230000, China; sa23174075@mail.ustc.edu.cn (Z.W.); shals@sibet.ac.cn (L.S.); ll19955544653@mail.ustc.edu.cn (L.L.); 2 Suzhou Institute of Biomedical Engineering and Technology, Chinese Academy of Sciences, Suzhou 215000, China

**Keywords:** continuum robots (CRs), variable stiffness, unknown confined space exploration, segmented constant curvature (SCC), scalability

## Abstract

Continuum robots (CRs) exhibit high compliance and environmental adaptability in confined, tortuous spaces, yet their inherent low stiffness and load capacity limit performance in precise positioning and stable support tasks. To solve the “soft-rigid” paradox, this study proposes and implements a three-segment tendon-driven variable-stiffness CR. Structurally, a segmented constant-curvature model directs the optimization of grid skeletons and notch parameters, enhancing bending consistency and motion predictability. Elongated flat airbag actuators, arranged in annular arrays, enable segment-level stiffness switching through the enhancement of surface properties like axial constraints and friction amplification. A time-sharing drive strategy decouples multi-segment coupling into sequential single-segment subproblems, reducing drivers and kinematic complexity while maintaining dexterity. Experimental results demonstrate that flexible-mode joints maintain near-constant curvature with stable motion (average end-effector trajectory error < 0.9 mm), and in rigid mode, stiffness increases by a factor of 5.77 (rated load: 4.0 N). Shape-locking disturbances during transitions are confined to millimeter levels (remote offset < 1.32 mm), with successful traversal of J/U/S-shaped and irregular paths confirmed in pipeline tests. This work introduces a practical, scalable system for designing variable-stiffness structures and enabling low-complexity multi-segment control, offering valuable insights for minimally invasive devices and industrial endoscopy in confined spaces.

## 1. Introduction

### 1.1. Background

Continuum robots (CRs), inspired by the biological structures of elephant trunks and octopus tentacles, offer remarkable adaptability and safety in unstructured environments. Their design—characterized by near-continuously distributed degrees of freedom and exceptional compliance—makes them especially effective in confined spaces. In contrast, rigid-link robots, with fixed link lengths and discrete joints, often have limited capability to traverse confined, tortuous passages, as they cannot readily conform to irregular boundaries [1,2]. These characteristics make CRs more valuable in various applications, including minimally invasive surgery [3], endoscopic inspection [4,5,6], disaster rescue, and industrial maintenance [7]. However, their high compliance also introduces inherent limitations in structural stiffness and load-bearing capacity [8,9]. In tasks requiring precise end-effector positioning, stable support, or loaded manipulation, insufficient stiffness often leads to end-point drift, morphological instability, and amplified control errors, significantly restricting further engineering applications of CRs [10].

To address this “soft–rigid” paradox, variable stiffness technology has emerged as a key research direction. An ideal CR should seamlessly switch between high dexterity/compliance and high stiffness/support, adapting dynamically to the demands of each task [11]. Common approaches to variable stiffness include granular and layer jamming [12,13,14,15], smart materials like shape-memory alloys (SMA) [16,17], and mechanical locking mechanisms [18,19]. Although these methods improve structural stiffness to some extent, they face significant challenges in terms of response speed, structural complexity, stiffness adjustment range, and miniaturization [7,20]. Although the bead-string-based jamming mechanism improves recoverability and stiffness enhancement through coordinated pneumatic control and organized particle arrangement, it can still suffer from limited controllability and stability due to particle accumulation and nonuniform distribution [21]. The SMA-based variable-stiffness continuum tube achieves substantial stiffening, yet thermal hysteresis and heat dissipation constraints lead to slow flexible–rigid transitions and hinder miniaturization [22]. The constrained-curvature mechanism with a redundant skeleton enables a high stiffness adjustment ratio and collaborative position–stiffness control [23]. However, its structural complexity makes it difficult to satisfy stringent size and integration requirements in applications such as minimally invasive procedure [19,24]. Therefore, achieving rapid, reliable, and adaptable stiffness adjustment within the constraints of miniaturization is a major challenge, particularly in confined-space applications.

Beyond structural stiffness, modeling and controlling CRs introduces further challenges. Thanks to their high degrees of freedom and nonlinear deformation characteristics, the forward and inverse kinematics of CRs rely on approximate models or nonlinear optimization techniques. In addition, Pourafzal proposes a piecewise constant strain kinematic model with high computational efficiency, but its accuracy degrades under heavy external loading [25]. By contrast, Shi builds on Cosserat rod theory and introduces a pressure-dependent dynamic modulus to address nonlinear behaviors, demonstrating significant advantages in accuracy and multi-mode control [26]. However, its complex model and numerous boundary conditions result in low computational efficiency. When extended to multi-segment serial structures, inter-segment coupling further increases solution dimensionality and computational cost, impairing real-time control and online planning performance [27]. To address this, integrating variable stiffness with simplified and scalable control systems for multi-segment robots is an essential research focus for their real-world application.

This study aims to develop a multi-segment continuum robot for exploration and manipulation in narrow, unknown environments. Specifically, we (i) design the robot skeleton to closely match a simplified kinematic model, enabling high computational efficiency while maintaining adequate accuracy; (ii) develop a compact variable-stiffness module that provides fast, stable, and wide-range stiffness modulation under miniaturization constraints to improve load-bearing capability; and (iii) integrate the stiffness mechanism with the actuation strategy to mitigate inter-segment coupling and reduce the computational cost of multi-segment control.

### 1.2. Contribution

This paper proposes and implements a three-segment tendon-driven CR with variable stiffness to address these challenges. By co-designing a ‘friction-dominated pneumatic locking stiffness mechanism’ alongside a ‘time-sharing actuation-based segmental decoupling strategy,’ we achieve a balance between dexterity, load capacity, and control complexity—ideal for applications in extended, unknown space exploration and manipulation. The main contributions of this paper are as follows:

Under the segmented constant-curvature modeling assumption, the grid-skeleton structure and key notch parameters are designed and optimized to improve bending consistency and end-point positioning accuracy [28].

A segmental friction-based variable stiffness module using flat airbag extrusion contact is proposed. Surface enhancements are introduced to improve axial constraint and interfacial friction, with reproducible locking performance metrics provided [3,29].

A time-sharing actuation control paradigm for multi-segment CRs is presented, transforming coupled control into sequential single-segment subproblem solving. This reduces control dimensionality and enhances system scalability, laying the foundation for real-time control [27].

### 1.3. Outline

This paper comprehensively expounds the design and research of a three-segment rope-driven variable-stiffness CR, focusing on the following aspects: Section 2 presents the overall integration scheme of the robot system and the robot’s driving strategy. Section 3 elaborates on the structural design method and manufacturing/assembly plan of the CR in depth. Section 4 proposes a parameter constraint model based on segmented constant curvature (SCC) and pneumatic friction locking. Section 5 puts forward a time-sharing actuation strategy, achieving multi-segment decoupling control through hardware architecture and state machine design. Section 6 verifies the system performance through hierarchical experiments, including key indicators such as motion accuracy, rigid-flexible switching deformation, stiffness adjustment range, load capacity, and pipeline traversal. Section 7 provides a detailed discussion. Section 8 summarizes the paper and points out future work.

## 2. System Overview

**Figure 1:**
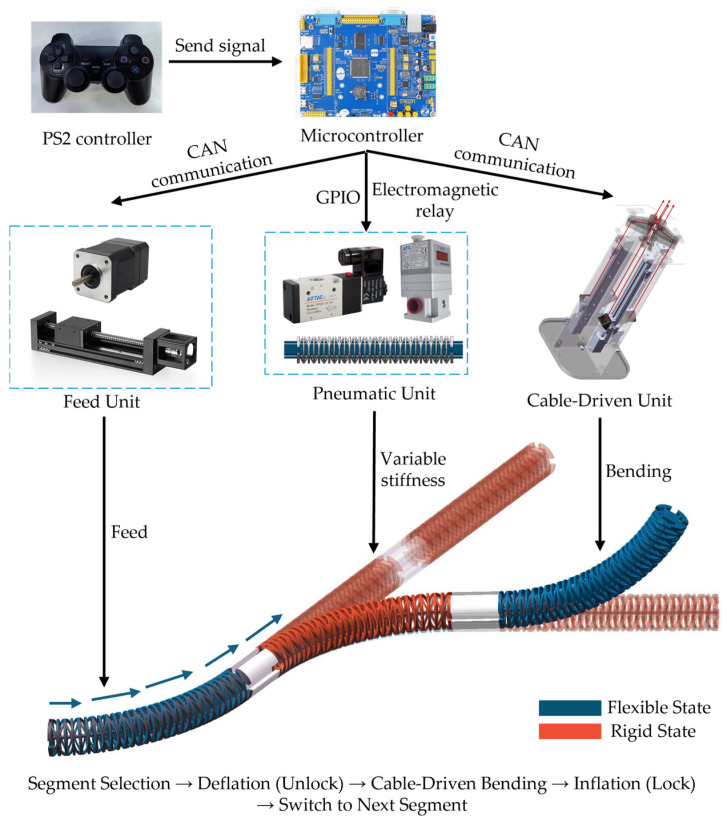
Continuum robot (CR) system.

As depicted in Figure 1, the CR system presented here consists of five key components: the robot body, rope-driven motion unit, feed unit, pneumatic variable-stiffness unit, and control system. The robot body comprises three modular continuum joints arranged in series, each offering omnidirectional bending capability. An annular array of flat airbags is strategically integrated around the perimeter of each joint, enabling segment-level rigid-flexible switching and ensuring shape locking. The rope-driven motion unit achieves bending formation of the target segment via tendon tensioning and release. The feed unit provides axial feed motion to adapt to the “push-form-lock” operation process in narrow spaces. The pneumatic unit adjusts the pressure of the airbags in each segment through proportional valves and solenoid valves, enabling controllable switching between flexible motion and rigid support.

The core workflow of the system follows a sequential “form-lock-advance” strategy: first, non-target segments are locked to form a rigid boundary; then, the target segment is depressurized, unlocked, and actuated by tendons to bend; finally, the segment is repressurized and locked to preserve its shape and increase stiffness. Overall advancement and pose transmission are then achieved via feed-in. This process decouples morphological generation and spatial traversal in multi-segment CRs into repeatable, segment-level operations, reducing the complexity of multi-segment coupled control implementation and offering a unified framework for task-level planning and experimental validation.

## 3. Mechanical Design and Fabrication

### 3.1. Modular Multi-Segment Body Design

**Figure 2:**
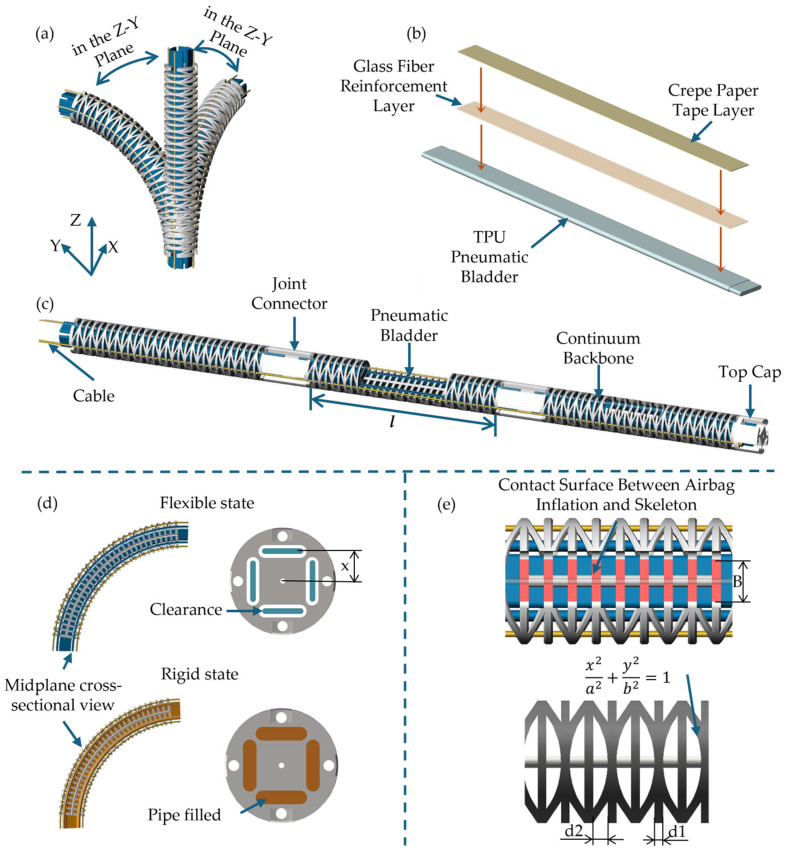
CR body; (**a**) schematic diagram of omnidirectional bending of continuum Joint; (**b**) manufacturing of the airbags; (**c**) three-segment CR and partial sectional view; (**d**) stiffness adjustment principle: inflation/deflation states of airbags in flexible and rigid states; (**e**) annotation of key parameters of continuum skeleton.

As depicted in Figure 2, the robot body comprises three modular continuum joints arranged in series. Each joint operates as an independent unit, providing omnidirectional bending capability and enabling rapid connection along with pneumatic-mechanical integration through inter-segment joints. This modular architecture not only meets the operational requirements of “local forming-whole advancing” in narrow spaces but also facilitates segment-level replacement and performance iteration in terms of manufacturing and maintenance.

The single-section skeleton incorporates a grid structure with a thin disk array and central axis. Disks are evenly distributed along the axial direction to provide radial support and guidance, while the central axis helps prevent axial slippage and non-uniform buckling under large bending, improving the consistency and predictability of the bending morphology. To better align with the segmented constant-curvature modeling assumption and reduce the abrupt bending changes caused by “local hinge-like behavior,” the skeleton introduces uniformly distributed elliptical notches as flexible units along the axial direction, making the bending more continuously distributed. As shown in Figure 2d, the variable-stiffness module comprises four axially arranged flat airbags embedded inside the continuum skeleton. In the unpressurized state, the airbags remain separated from the inner wall and can slide during bending; the overall stiffness is primarily determined by the skeleton. In the pressurized state, the airbags expand and press against the inner wall of the skeleton, generating friction that resists relative sliding and stabilizes the curvature, thereby increasing stiffness. The stiffness can be continuously tuned by regulating the inflation pressure. As illustrated in Figure 2b, modifying the airbag surface/geometry further enhances the frictional locking effect, as discussed in Section 3. Meanwhile, drive rope channels and tendon sheaths are arranged in the circumferential direction of the cross-section to reduce friction and wear and enhance tension transmission stability. The joint connector serves dual functions: structural connection and end positioning. Firstly, it ensures the coaxial alignment and repeatable assembly of the three-segment series connection. Secondly, it provides a secure installation for the variable-stiffness airbag, minimizing the risk of leakage and loosening during long-term operation.

### 3.2. Key Parameter Design and Notch Sizing

To meet the narrow-space operational requirements for form factor, flexible motion, segment-level rigid-flexible switching, and manufacturability, this study uses a constraint-driven parametric design approach to determine the key geometric parameters and notch dimensions of a single-segment joint. The relevant geometric parameter definitions are shown in Figure 2, and the core constraints are summarized in Table 1.

Based on the constraints in Table 1, a constraint-driven parametric design process is used to determine the geometric dimensions of the single segment and notch. The steps are as follows:

Step 1 (Scale Sizing): Given the allowable passage diameter $D_{req}$ specified by the task scenario, determine the maximum outer envelope diameter D of the robot, verify the outer diameter constraint, define the length-to-diameter ratio, and provide the initial scale of the single-segment shape and inter-segment connection.

Step 2 (Cross-Section Layout and Manufacturability Check): On the established outer diameter, complete the layout of cross-sectional holes (including drive rope ducts, air passages, etc.), and verify manufacturability and assemblability using the minimum feature size constraint $w_{min}\geq w_{poc}$. Iteratively adjust hole dimensions, wall thickness, and layout spacing if necessary.

Step 3 (Notch Parameter Screening and Integrated Check): Screen and combine notch geometric parameters under the premise of satisfying the cross-sectional layout, ensuring they simultaneously meet the kinematic performance constraints ($\theta \geq \theta_{0},\;R\leq R_{0}$) and the airbag/joint accommodation and interference check under the maximum bending posture. Among these, locking reliability is treated as a key verification condition for rigid-state load-bearing: under the most unfavorable working conditions, the locking segment must meet the anti-slip threshold to guarantee shape retention and support capability after rigid-flexible switching. The anti-slip criterion involves modeling and calibration of contact pressure, contact area, and friction parameters, which constitutes a core component of locking mechanism research and characterization. The mathematical model and experimental verification of these criteria are further detailed in Section 4 and Section 5 of this paper.

### 3.3. Fabrication and Assembly

The manufacturing of the robot body comprehensively considers the requirements of compliance, wear resistance, and geometric accuracy: the single-segment skeleton is formed integrally by Selective Laser Sintering (SLS) process using TPU material to achieve high elasticity and complex inner cavity forming capability; the inter-segment joint is printed with Stereolithography (SLA) resin to ensure assembly accuracy and connection stiffness, and is reliably connected to the skeleton via adhesive bonding. The drive rope and tendon sheath are threaded and pretension-calibrated after the skeleton is formed to ensure that the tension adjustment range covers the maximum bending requirement of a single segment.

The airbag module is manufactured via TPU hot pressing, followed by gas leak inspection post-forming. It is then assembled and fixed to the skeleton/joint at the designed position; subsequently, Airbag Axial and Surface Enhancement (AASE) is implemented: glass fibers are pasted along the axial direction to suppress undesired expansion, and the surface material is treated to improve the interface friction condition, as shown in Figure 2b. Finally, the air duct connection and sealing are completed.

The overall assembly process of the robot can be summarized as follows: (1) Assembly and positioning of the single-segment skeleton and joint; (2) Airbag installation, enhancement, and airtightness testing; (3) Series assembly of three segments and system-level leakage testing; (4) Threading and calibration of drive ropes and tendon sheaths; (5) Integration with external rope-driven/pneumatic/feed units and joint commissioning.

## 4. Mechanism and Modeling

### 4.1. Segmented Constant-Curvature Approximation and Notch Geometry

To simplify the structural dimension design and control strategy, this paper approximates the bending morphology of a single-segment continuum joint in its flexible state as a constant-curvature arc. This approximation, widely used in CR research [30,31], relies on the uniform distribution of compliant elements along the axial direction and the consistency of axial and radial deformation. Considering the “uniform notch array” feature of the joint, the single-segment bending can be described by two variables: the bending angle θ  and the radius of curvature R, which follow the geometric relationship.

(1) $$R=\frac{L}{\theta }$$

L is the arc length of the segment when bent.

Notch Geometry Constraint on Bending Capability. Given the notch geometric parameters (as shown in Figure 2c,e), the achievable bending angle θ of the joint is given by:

(2) $$\theta \;=\;\frac{2\;(2-\sqrt{2})\mathrm{bl}}{(d_{1}\;+\;d_{2})D}$$

where $d_{1}$ is the axial characteristic thickness of the segment related to the “solid segment” (the thickness of the disk); $d_{2}$ is the axial geometric spacing of the segment related to the “notch segment”; b  is the minor axis length of the elliptical notch; l  is the length of the continuum joint.

In the structural design phase, θ and R  must satisfy the target scenario’s capability indicators for the “maximum bending angle” $\theta_{0}$ and “minimum bending radius” $R_{0}$:

(3) $$\theta \;>{\;\theta }_{0}$$

(4) $$R<R_{0}$$

Furthermore, to enhance the consistency of constant curvature and reduce local “hinge-like” bending, the axial pitch of the compliant unit should be minimized. Specifically, $\left(d_{1}\;+\;d_{2}\right)$ should be reduced while meeting manufacturing and strength constraints.

### 4.2. Pneumatic Friction Locking Mechanism and Anti-Slip Criterion

In the rigid state, the flat airbag inflates and expands to form an extrusion contact interface with the inner wall of the skeleton, thereby generating normal extrusion force and enhancing the friction capacity of the interface. This process can be equivalently modeled as: the airbag exerts a normal pressure on the contact interface under the gauge pressure P, and the maximum frictional resistance provided by the interface is related to the contact area and friction coefficient. To ensure locking reliability during structural design, the paper examines the “most unfavorable slip condition”, where the joint end is most susceptible to slip when the continuum bending angle θ = 0° and the external load direction is perpendicular to the airbag’s wide plane. Using the moment balance and equivalent friction model, the anti-slip condition is derived.

(5) $$4\frac{d_{1}}{d_{1}+d_{2}}\mathrm{bP}\mu x\;>\;F$$

where F  is the equivalent load at the joint end; P  is the input air pressure of the airbag; μ  is the equivalent friction coefficient between the outer surface of the airbag and the inner wall of the skeleton (the equivalent friction coefficient μ between the airbag outer surface and the TPU skeleton was obtained using a sliding-friction test following [32]. Briefly, the TPU specimen was fixed horizontally, the airbag surface material was placed on it under a known normal load, and the material was pulled at a constant rate. The friction coefficient was computed as μ =F/N , where F is the measured steady-state tangential force and N is the applied normal force.); b is the equivalent width of the contact surface (corresponding to the effective coverage length of the airbag); the coefficient $\frac{d_{1}}{d_{1}+d_{2}}$ is the effective contact ratio of the unit length; x is the distance from the centroidal axis of the continuum to the middle plane of the airbag.

Equation (5) presents the design relationship between pressure, friction, and load-bearing. Given the external load requirement F and structural parameters ($d_{1},d_{2},b,l$), the minimum locking pressure $P_{min}\;$ can be derived, guiding the selection of the pneumatic system and the setting of safety margins. (This criterion is cited as the “locking slip constraint” in Section 3.2).

Maximum bending airbag coverage and joint length allowance. To ensure full coverage of the continuum joint during bending and compatibility with the joint connector, the allowable length of the airbag must be reserved. According to the constant-curvature geometric relationship, the required reserved length can be estimated by the following formula:

(6) z = 2θx+c

where  z is the extra airbag length required to be accommodated by the joint (or cavity); c is the manufacturing and assembly error allowance, used to enhance assembly robustness and avoid interference and sealing failure under the maximum bending configuration.

### 4.3. Finalization of Robot Key Parameters

**Figure 3:**
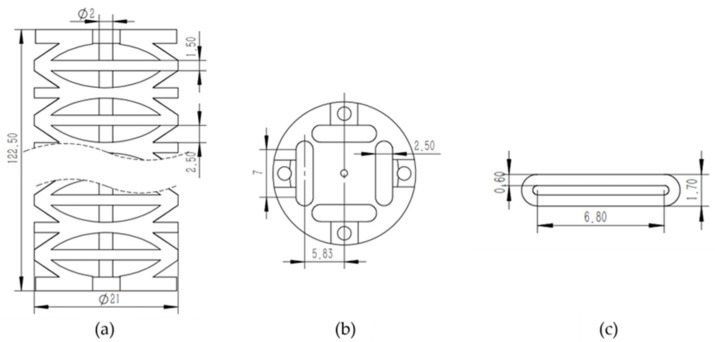
Continuum joint configuration and key parameters; (**a**) axial schematic and dimensions; (**b**) cross-sectional schematic and dimensions; (**c**) airbag cross-sectional dimensions.

Following the parameter-design procedure described in Section 3, we selected an outer diameter of 21 mm to meet the constraints of confined-space exploration. Considering the minimum manufacturing tolerance and the required internal channels, the cross-sectional layout and the airbag cross-sectional dimensions were determined (Figure 3b,c). Based on the target application and flexibility requirements, the minimum bending radius per segment was set to 50 mm and the maximum bending angle to 120°. With these specifications, we formulated the design constraints using Equations (2)–(5), together with bending strength constraints of the continuum joint. Under the constant-curvature bending requirement, we performed an optimization search to identify an optimal parameter set. After rounding to manufacturable values, the final joint thickness and notch parameters were obtained, and the corresponding axial dimensions are shown in Figure 3a. This parameter set provides a reproducible basis for fabrication/assembly, modeling of the locking mechanism, and implementation of the time-sharing actuation strategy, and it was used for the experimental validation in Section 6.

## 5. Actuation and Control

### 5.1. Hardware System Composition and Signal Flow

**Figure 4:**
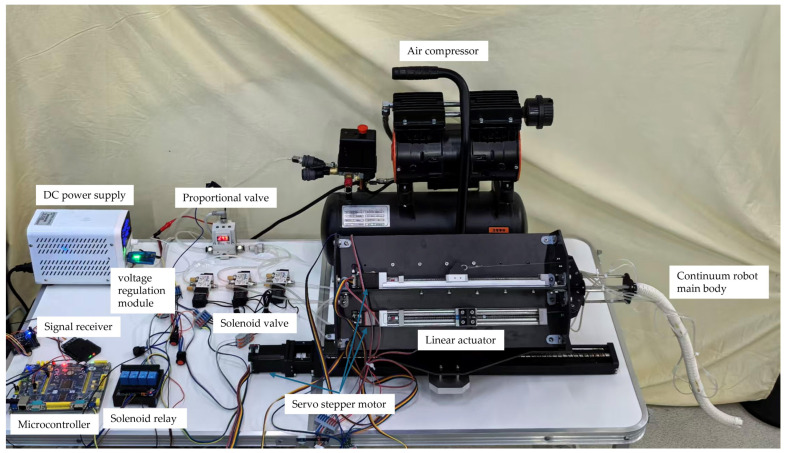
Control and drive hardware configuration.

The experimental platform comprises three subsystems: a rope-driven, pneumatic stiffness-variable, and feed subsystem. The hardware configuration of the system is shown in Figure 4. The power supply and main control unit are composed of a DC power supply (GPS305D, WANPTEK, Shenzhen, China) and a single-chip microcomputer (SCM, STM32F103ZET6, STMicroelectronics, Geneva, Switzerland). The SCM is responsible for coordinating and scheduling motor and valve control signals, and provides a human–machine interaction interface to achieve remote control and state switching of the entire machine.

The rope-driven subsystem enables omnidirectional bending of the continuum joint with four linear drive units, each consisting of a ball screw guide rail (FSK30J, FUYU, Shenzhen, China) and a 28-stepper motor (28DZ40, ZDT, Shaoxing, China). Rope length variation is controlled via CAN communication, generating differential tension to drive the bending. In the framework of this paper, the rope-driven subsystem mainly undertakes the function of “forming the flexible segment,” and its control variables can be represented by rope length increments or equivalent bending parameters (such as bending angle and bending direction).

The pneumatic variable-stiffness subsystem enables segment-level rigid-flexible switching and stiffness adjustment. The system includes an electromagnetic relay (833H-1C-C, Song Chuan, Shanghai, China), air compressor (1600W-30L, OTS, Ningbo, China), proportional valve (ITV1030-312BL, SMC, Tokyo, Japan), and three-way solenoid valves (VT307-5G1-01, SMC, Tokyo, Japan). The compressor supplies air, which is divided into three paths through a Y-type tee. The air then passes through the relay-controlled solenoid valve (turned on/off by the microcontroller GPIO) and the adjustable pressure module/proportional valve, and finally enters the airbags of each segment to realize airbag inflation/deflation and pressure adjustment, thereby completing the switching between flexible/rigid states and adjusting the locking support capability.

The feed subsystem consists of a 42-stepper motor (42ZDT60A, ZDT, Shaoxing, China) and a ball screw guide rail (FSK40J, FUYU, Shenzhen, China), which provides axial feed capability to enable the robot to perform “feed-form-lock” sequential operations in narrow spaces.

The system is remotely controlled using a PS2 joystick (Sony, Tokyo, Japan), enabling the operator to coordinate rope drive, air pressure, and feed commands. The overall control block diagram of the system is shown in Figure 1.

### 5.2. Time-Sharing Actuation Strategy and State Machine Description

Driving multiple segments simultaneously in multi-segment CRs increases the complexity of solving high-dimensional coupled inverse kinematics. To improve engineering implementability, we propose a time-sharing actuation strategy [33] where only the “unlocked segment” is driven at any given time, while other segments are locked by airbag inflation, providing stable boundary conditions and reducing inter-segment coupling [34].

**Figure 5:**
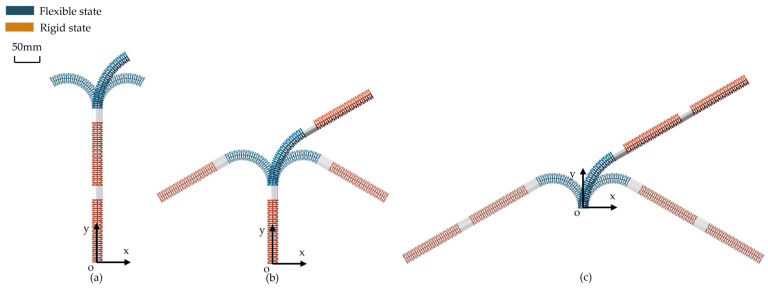
States of each segment joint during robot motion; (**a**) state when controlling the end segment; (**b**) state when controlling the middle segment; (**c**) state when controlling the proximal segment.

The robot consists of three segments connected in series, and the inflation/deflation states of the continuum joint during motion are shown in Figure 5. If it is necessary to control the nth continuum joint to bend, the airbag of the nth segment is deflated to enter the flexible state, while the airbags of the other two segments are inflated to enter the rigid (locked) state. The bending formation and attitude adjustment of the nth segment are primarily achieved through rope drive. Based on this, the three-segment system can be summarized into three repeatable control states:

S1: Lock segments 2 and 3, control segment 1;

S2: Lock segments 1 and 3, control segment 2;

S3: Lock segments 1 and 2, control segment 3.

In each state, the locked segments provide an approximate rigid boundary support, allowing the controlled segments to undergo bending formation in a lower-dimensional space; state switching is achieved through valve-controlled inflation/deflation, enabling the system to perform task-level coordination between “flexible deformation” and “rigid support.”

For multi-segment CRs, due to their high degrees of freedom and strong inter-segment coupling, the fully driven approach of “simultaneous driving of multiple segments” typically requires solving the inverse kinematics in the form of high-dimensional nonlinear optimization. The computational complexity increases combinatorially with the number of segments, directly restricting real-time control and online planning capabilities. The time-sharing actuation strategy transforms the high-dimensional coupled problem of “simultaneous driving “ into serial subproblems: only one segment’s degrees of freedom are activated at a time, while others are locked as fixed boundary conditions. Thus, the mapping from the configuration space to the drive space can be solved step-by-step for each segment, and the overall computational cost is the superposition of the costs of each subproblem, rather than the combinatorial increase in multi-segment coupled solving. Meanwhile, compared to the fully driven scheme where each segment is equipped with 4 ropes and driven simultaneously, this strategy significantly reduces the number of drive motors, lowers the hardware complexity and system debugging difficulty, and improves the scalability of the system.

The availability of this strategy is evaluated in Section 6 through locking the distal offset, stiffness-pressure mapping, and task-level experiments.

## 6. Experiments and Results

This chapter verifies three core claims of this paper: (i) the single segment exhibits approximate constant curvature and good repeatability in the flexible state; (ii) pneumatic friction locking enables significant stiffness variation and enhances load-bearing capacity, with controllable disturbance during rigid-flexible switching; (iii) time-sharing actuation is applicable in narrow and complex pipeline tasks. All experiments were conducted on the existing platform. An NDI optical tracking system was used to record the trajectories of the end-effector marker points [35], enabling a quantitative comparison between the unprocessed airbag group and the AASE group. For some experiments, the end of the continuum joint in a rigid horizontal state must withstand a 2N load without airbag slippage. Combining this with Equation (5), the minimum locking pressure of the airbag is calculated to be greater than 245 kPa. Through testing, the maximum locking pressure at which the prepared airbag does not leak gas is 300 kPa. To ensure the stability and functionality of the airbag during the experiment, a locking pressure of 250 kPa (close to the lower limit) is selected, and the state of the continuum joint at this pressure is regarded as the rigid state.

### 6.1. Constant-Curvature Bending Characteristics and Motion Repeatability

**Figure 6:**
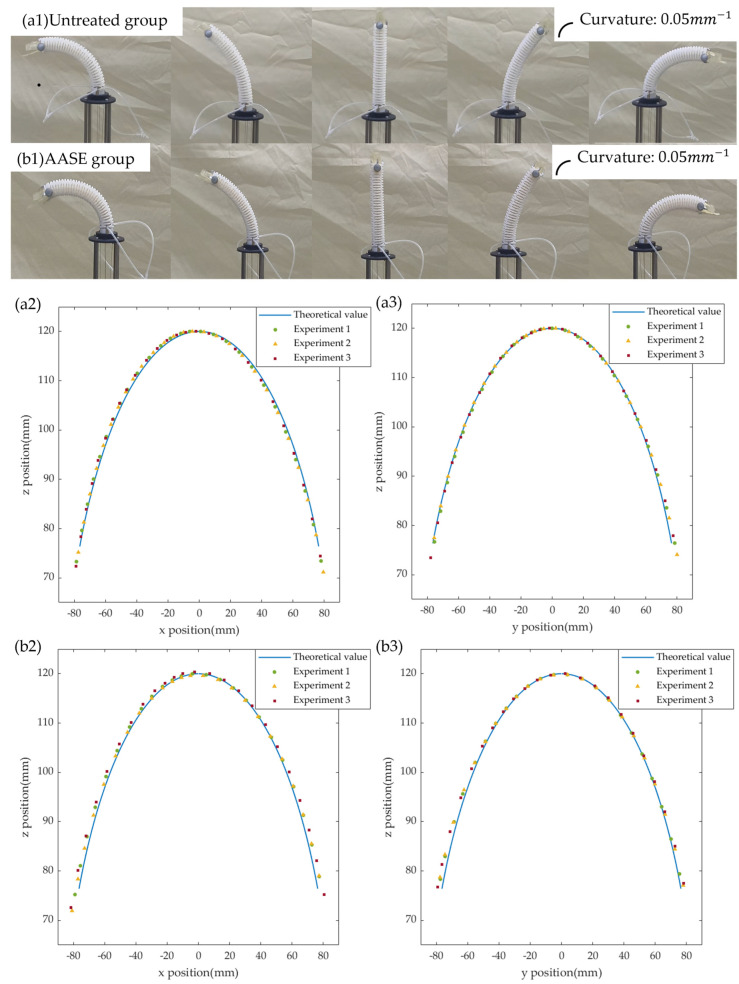
Bending curvature characteristics and motion stability experiment; (**a1**) experimental process of the untreated group; (**a2**) end trajectory of the untreated group in the x-z plane; (**a3**) end trajectory of the untreated group in the y-z plane; (**b1**) experimental process of the airbag axial and surface enhancement (AASE) group; (**b2**) end trajectory of the AASE group in the x-z plane; (**b3**) end trajectory of the AASE group in the y-z plane.

To evaluate the constant curvature and motion repeatability of the single joint in the flexible state, we used the NDI optical positioning system to track the end mark points. Reciprocating bending tests were performed within the joint’s bending range of [−90°, 90°], repeated three times under the same drive command to assess repeatability. During the experiment, the measured end trajectory is compared with the theoretical trajectory based on the constant-curvature assumption, and the trajectory comparison and experimental process are shown in Figure 6 For ease of quantitative evaluation, the positioning errors of the end trajectory in the x–z plane and y–z plane are statistically analyzed, and the results are shown in Table 2. End positioning error of the untreated and AASE groups in the flexible state; the repeatability error statistics are shown in Table 3.

The results show that the average errors of the unprocessed airbag group in the x–z/y–z planes are 0.591 and 0.317 mm, respectively, while the AASE group’s errors are 0.888 mm and 0.622 mm. The slightly larger end-effector positioning error observed in the AASE group, relative to the baseline (untreated) group, is likely due to the airbag surface enhancement, which can alter the constant-curvature bending behavior of the continuum joint. Nevertheless, the errors remain within an acceptable range, corresponding to 0.725% and 0.508% of the segment length in the x–z and y–z planes, respectively. In terms of repeatability, both groups show trajectory overlap errors of less than 1.1 mm. The joint exhibits stable constant-curvature bending and high motion consistency in the flexible state, confirming the validity of the “structural design—constant-curvature assumption—end trajectory prediction” model and providing a reliable segment-level basis for subsequent drive and control.

### 6.2. Rigid-Flexible Switching Shape Locking Performance

**Figure 7:**
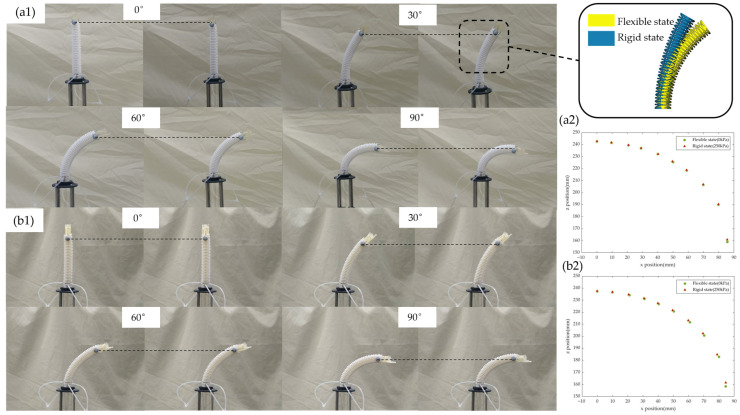
Experimental investigation of shape locking performance during flexible-rigid transition: (**a1**) bending morphology of the untreated group before and after pneumatic inflation; (**a2**) distal positions of the untreated group under different bending angles in the two states; (**b1**) bending morphology of the AASE group before and after pneumatic inflation; (**b2**) distal positions of the AASE group under different bending angles in the two states.

A core assumption of the time-sharing actuation strategy is that locked non-active segments should exhibit approximate rigid support during the switching process, without introducing significant end disturbances. To verify this, the ‘distal offset’ is defined as the shape-locking error during rigid–flexible switching. A 0–90° bending cycle (10° increments) was performed. At each preset angle, pressure release and reinflation (to 250 kPa) were repeated three times with a 3 s interval. The end-effector marker position before and after switching was recorded, and the distal offset was computed as the Euclidean distance between the two positions. The reported values are averages over the three repetitions. Morphological comparison of the two sets of joints under flexible/rigid states and the end-effector position distribution at different angles are shown in Figure 7.

The results show that the average distal offset of the untreated group is 0.565 mm (0.471% of the total length), while that of the AASE group is 1.32 mm (1.10% of the total length). Both groups exhibit only small positional variations, indicating that the shape disturbance induced by airbag inflation/deflation remains within the millimeter range and below the conservative permissible error bound (5.6% of the segment length) [36]. This result is consistent with the theoretical expectation that “friction locking inhibits relative slippage → curvature maintenance” is effectively implemented, and provides key evidence for the rigid-flexible switching shape locking mechanism.

### 6.3. Relationship Between Bending Stiffness and Air Pressure

**Figure 8:**
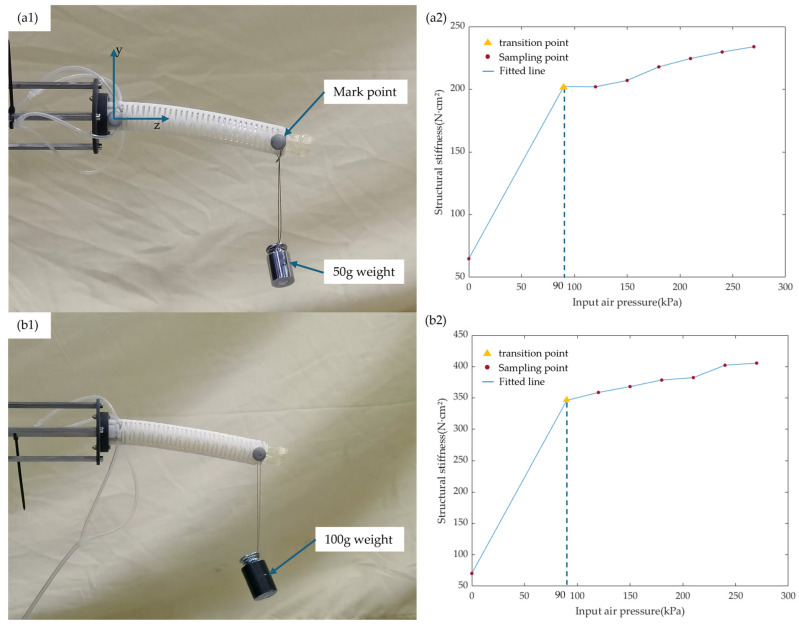
Structural bending stiffness test experiment: (**a1**) experimental diagram of cantilever beam bending stiffness test for the untreated group; (**a2**) experimental results of the untreated group; (**b1**) experimental diagram of cantilever beam bending stiffness test for the AASE group; (**b2**) experimental results of the AASE group.

To quantify the variable stiffness performance and establish a stiffness-pressure mapping relationship applicable to control, this paper estimates the equivalent bending stiffness EI of the joint using the Cantilever Beam Deflection Method. Stepwise pressurization tests were conducted for the baseline (untreated) and AASE groups from 0 to 270 kPa (30 kPa per step). A constant load of 50 g/100 g was applied at each pressure point, and each condition was tested in triplicate. The end displacement was recorded and averaged, and the bending stiffness was then calculated using the cantilever-beam deflection formulation. The experimental setup and EI−P  curve results are shown in Figure 8.

The results exhibit a distinct two-stage pattern: during the initial phase (air pressure < 90 kPa), the stiffness increases rapidly with air pressure, dominated by the expansion of the airbag leading to an increase in effective contact area; beyond this threshold (air pressure ≥ 90 kPa), the stiffness growth slows down, primarily driven by the enhancement of contact normal force as the contact becomes more fully developed. In both flexible and rigid states, the stiffness of the untreated group is 64.82 $N\cdot m^{2}$ and 233.91 $N\cdot m^{2}$ (magnification: 3.61), respectively, while that of the AASE group is 70.32 $N\cdot m^{2}$ and 405.48 $N\cdot m^{2}$ (magnification: 5.77). These results demonstrate that air pressure enables continuously adjustable stiffness with improved magnification, aligning with the proposed contact-friction locking mechanism. The low-pressure stage corresponds to “contact area expansion,” whereas the high-pressure stage corresponds to “normal force enhancement.” This supports the trend that “Higher air pressure leads to higher friction force, resulting in higher load-bearing capacity and anti-slip margin” in the locking model.

### 6.4. Quantification of Load-Bearing Capacity

**Figure 9:**
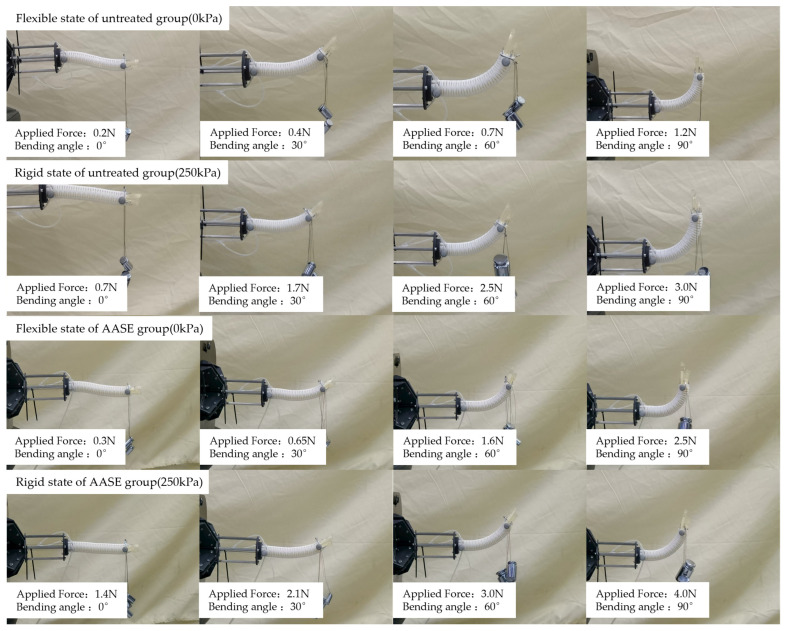
Quantitative experimental diagram of load-bearing capacity for untreated and AASE groups.

**Figure 10:**
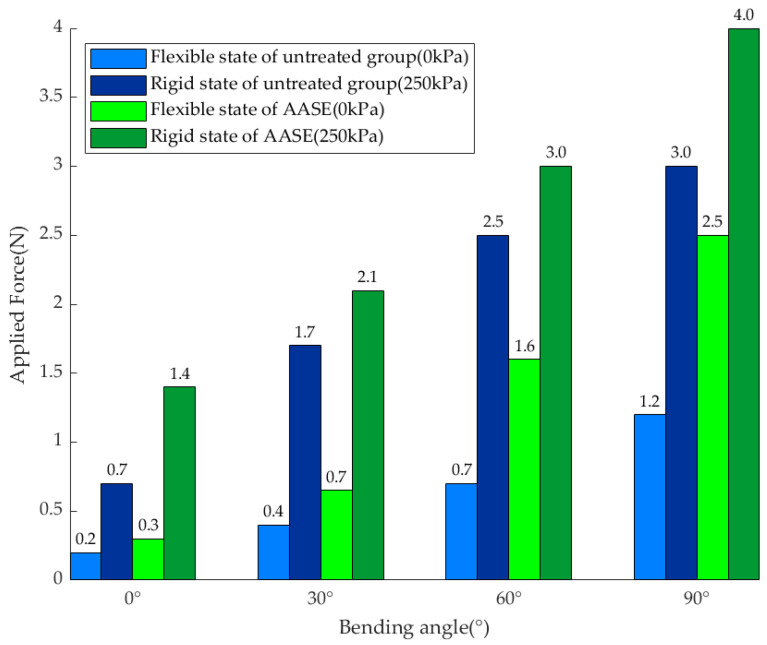
Quantitative experimental results of load-bearing capacity.

To assess the engineering significance of stiffness modulation, the load-bearing capacity of the flexible state (0 kPa) and rigid state (250 kPa) was tested at bending angles of 0°, 30°, 60°, and 90°. Each condition was repeated three times. The rated load capacity was defined using the criterion that the end deviation does not exceed 10% of the segment length [3]. Therefore, the experiment adopted a stepwise loading method (with a step size of 2 g) to apply loads to the end of the joint, and “the end deviation not exceeding 10% of the total length of the joint (12 mm)” was used as the rated load-bearing criterion. The experimental setup is shown in Figure 9, and the summarized results of the rated load-bearing capacity are presented in Figure 10.

The results show that the rated load-bearing capacity of the untreated group in the flexible/rigid states is 1.2 N/3 N, and that of the AASE group is 2.5 N/4.0 N, with a significant improvement trend observed under different postures. Notably, the rigid-state load capacity of both the baseline (untreated) group and the AASE group is higher than that in the flexible state. Moreover, for both groups, the rated load capacity increases with bending angle in both actuation states. This indicates that the improvement in stiffness ratio can be further converted into a gain in available load-bearing capacity, verifying the performance transmission chain of “pneumatic locking → stiffness improvement → enhanced anti-deformation capability under external loads”. Meanwhile, the improvement in load-bearing capacity under multiple postures also indirectly supports that the interface anti-slip capability in the locked state meets expectations, providing a mechanical basis for the availability of the “locked support segment” in mission-level pipeline crossing tasks.

### 6.5. Pipeline Crossing Mission Validation

**Figure 11:**
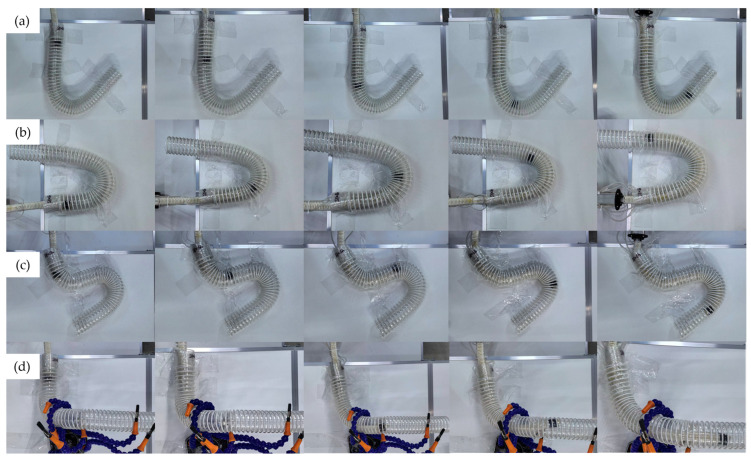
Pipeline traversal experimental process; (**a**) J-type pipeline; (**b**) U-type pipeline; (**c**) S-type pipeline; (**d**) spatial irregular pipeline.

To verify the system’s applicability in narrow and curved spaces, the configuration uses two AASE groups (with stronger load-bearing capacity) at the front and one untreated group (with higher flexibility) at the end. Crossing tests were conducted in J/U/S and spatial irregular configurations built with a 45 mm inner diameter corrugated pipe (PVC-45, YZ, Ningbo, China) [37]. The robot’s crossing process in different pipe configurations is shown in Figure 11.

The experimental results demonstrate that the robot can successfully traverse all test pipelines: in the flexible state, it can smoothly adapt to pipeline bending and achieve passage; after switching to the rigid state, it can quickly lock its shape and provide stable support for the flexible end; during traversal, the extrusion on the pipe wall is minimal. This mission validation forms a closed loop with the segment-level experiments: the constant-curvature consistency of the segments ensures the predictability of motion during traversal; the small disturbance of locking switching guarantees the stability of state transition; the improvement in stiffness and load-bearing capacity provides the mechanical basis for the “locked support segment” in mission-level pipeline crossing tasks, thereby reflecting the synergistic advantages of “flexible traversal and rigid support”.

## 7. Discussion

Improving motion accuracy in continuum robots (CRs) commonly relies on nonlinear optimization or high-fidelity continuum modeling (e.g., Cosserat-rod formulations) to better capture deformation behaviors [26]. However, such models typically introduce substantial computational complexity and numerous boundary conditions, which can hinder real-time implementation—particularly for multi-segment systems. In this work, we adopt a parameter-optimization-based skeleton design that enables the robot to closely approximate a piecewise constant-curvature kinematic model. This strategy reduces computational burden while maintaining adequate accuracy for real-time control. In addition, the proposed time-sharing actuation strategy decomposes the high-dimensional, coupled actuation problem of a multi-segment CR into a sequence of segment-wise subproblems, thereby reducing implementation complexity and improving practical controllability.

With respect to variable stiffness, compared with representative mechanisms such as particle-jamming-based approaches [21], the proposed stiffness module—based on a circular arrangement of elongated strip airbags—offers a mechanically simpler implementation and supports rapid, repeatable stiffness modulation with stable response characteristics. Furthermore, relative to baseline airbag-based designs [3], the proposed airbag axial and surface enhancement (AASE) approach enhances frictional locking at the airbag–skeleton interface and expands the achievable stiffness modulation range. Together, these design choices address a common trade-off in confined-space CR applications, namely high flexibility versus low stiffness/load-bearing capability, by enabling a “flexible traversal–rigid support” operating paradigm.

## 8. Conclusions

This study proposes and implements a three-segment tendon-driven CR system with a compact variable-stiffness module for operation in narrow and complex environments. A parameter optimization scheme is introduced to synthesize continuum-joint designs tailored to different constraints, offering scalability across scenarios. The effectiveness of the integrated structural design, stiffness mechanism, and control strategy is validated through hierarchical experiments.

Experimental results show that, in the flexible state, the end-effector trajectory error remains small and repeatable, on the order of millimeters. In the rigid state, bending stiffness increases by up to 5.77× (see Figure 8), resulting in a marked improvement in load-bearing capacity. During rigid–flexible switching, the end-shape disturbance remains on the order of millimeters, indicating that the locking mechanism provides acceptable shape retention under frequent transitions. In addition, pipeline traversal experiments demonstrate stable locomotion in representative configurations, including J-type, U-type, S-type, and spatially irregular pipelines. These results highlight the practical benefit of combining flexible traversal with rigid support for confined-space operations.

Despite the demonstrated applicability of the proposed system, several aspects warrant further improvement, including friction attenuation at the stiffness interface over prolonged operation, airbag durability, and online compensation for error accumulation in multi-segment connections. Future work will focus on (i) evaluating the long-term reliability and service life of the variable-stiffness module, (ii) optimizing closed-loop compensation control for segment-to-segment error accumulation [38], and (iii) further integrating the pneumatic supply and valve control subsystems to support miniaturization. These efforts are expected to broaden the system’s applicability to narrow-space scenarios such as minimally invasive instruments, industrial endoscopy, and pipeline inspection.

## Figures and Tables

**Table 1:** Summary of key design constraints for continuum joints.

Constraint Categories	Constraint Items	Constraint Purpose/Meaning	Typical Expression or Verification Method
Scale and Manufacturing	Outer Diameter Constraint	Meet the requirements of target channel entry/passing	Check by comparing with the size of the task channel/opening and take the maximum envelope
Scale and Manufacturing	Minimum Feature Size	Ensure manufacturability and assembly of holes/channels	Check against the minimum size limit of the process (bore diameter/wall thickness/narrowest notch, etc.)
Kinematic Performance	Maximum Bending Angle	Meet attitude reachability	Calculate using constant-curvature geometric relationship and verify if it meets the target value (refer to Section 4 for criteria and parameter calibration)
Kinematic Performance	Minimum Bending Radius	Meet the requirement of passing through narrow spaces	Calculate using constant-curvature geometric relationship and verify if it meets the target value (refer to Section 4 for criteria and parameter calibration)
Integration and Interference	Airbag/Joint Accommodation	No interference and sealable under maximum bending	Perform interference check under maximum bending posture and verify length reservation (refer to Section 4 for criteria and parameter calibration)
Morphological Consistency	Approximate Constant Curvature	Reduce local “hinge-like” bending	Apply constraints on unit segment pitch and notch uniformity to avoid local “hinge-like” bending (refer to Section 4 for criteria and parameter calibration)
Locking Reliability	Locking Without Slippage	Ensure stable bearing under rigid state	Verify anti-slippage threshold (refer to Section 4 for criteria and parameter calibration)
Structural Mechanics	Maximum Bending Angle	Meet attitude reachability	Perform strength and stiffness verification under pushing/contact conditions (FEA/experimental validation if necessary)

(1) Let $D_{max}$ denote the maximum outer envelope diameter of the robot, and $D_{req}$ be the maximum allowable diameter specified by the task scenario. The outer envelope diameter constraint is defined as: $D_{max}<D_{req}$. (2) Let $w_{min}$ represent the minimum feature size of the robot, including (but not limited to) the diameter of the rope duct, the width of the air passage, the thickness of the thin wall, and the narrowest width of the notch. Let $w_{poc}$ be the minimum achievable size limit of the manufacturing process (determined by the specifications of SLS/SLA equipment or calibration tests). The minimum feature size constraint is: $w_{min}>w_{poc}$ This constraint aims to ensure the manufacturability and assembly feasibility of the robot, and to mitigate the impacts of channel blockage, thin-wall defects, and dimensional deviations on its performance. (3) The constraints for the maximum bending angle and minimum bending radius are, respectively: $\theta \geq \theta_{0}$, $R\leq R_{0},$ where θ  (bending angle) and R (bending radius) are calculated using the constant-curvature geometric relationship. The expressions for θ  and R, as well as the definitions of their variables, are provided in Chapter 4. (4) Through finite element simulation (ANSYS Workbench 2022 R1), the maximum equivalent stress at the maximum bending angle of the rigid joint is determined. The structural strength constraints can be expressed as: $\sigma_{max}\leq \left[\sigma \right].$

**Table 2:** End positioning error of the untreated and AASE groups in the flexible state.

Treatment Method	Untreated Group		AASE Group	
Measurement Error Plane	X-Z Plane	Y-Z Plane	X-Z Plane	Y-Z Plane
Maximum Error	0.981	1.849	2.389	2.273
Mean Error	0.591	0.317	0.888	0.622

**Table 3:** Trajectory error of repeatability experiment for untreated and AASE groups.

Treatment Method	Untreated Group	AASE Group
Maximum error	0.551	0.899
Mean error	0.238	0.337

## Data Availability

The original contributions presented in this study are included in the article. Further inquiries can be directed to the corresponding author.

## Abbreviations

The following abbreviations are used in this manuscript:

AASE	Airbag axial and surface enhancement
CR	Continuum robot
SCC	Segmented Constant Curvature
SLS	Selective Laser Sintering
SLA	Stereolithography
SMA	Shape-memory alloys
$D_{max}$	The maximum outer envelope diameter of the robot
$D_{req}$	The maximum allowable diameter specified by the task scenario
$w_{min}$	The minimum feature size of the robot
$w_{poc}$	The minimum achievable size limit of the manufacturing process
θ	Bending angle
R	Bending radius
L	The arc length of the segment when bent
$d_{1}$	The axial characteristic thickness of the segment related to the “ solid segment”
$d_{2}$	The axial geometric spacing of the segment related to the “ notch segment”
b	The minor axis length of the elliptical notch
l	The length of the continuum joint
$\theta_{0}$	Maximum bending angle
$R_{0}$	Minimum bending radius
F	The equivalent load at the joint end
P	The input air pressure of the airbag
μ	The equivalent friction coefficient between the outer surface of the airbag and the inner wall of the skeleton
b	The equivalent width of the contact surface
$\frac{d_{1}}{d_{1}+d_{2}}$	The effective contact ratio of the unit length
x	The distance from the centroidal axis of the continuum to the middle plane of the airbag
$P_{min}$	The minimum pressure
z	The extra airbag length required to be accommodated by the joint
c	The manufacturing and assembly error allowance
$\sigma_{max}$	The maximum equivalent stress at the maximum bending angle of the rigid joint
$\left[\sigma \right]$	Allowable Safety Strength of Selected Materials
